# Inhibition of miR-34a-5p alleviates hypoxia-reoxygenation injury by enhancing autophagy in steatotic hepatocytes

**DOI:** 10.1242/bio.033290

**Published:** 2018-03-15

**Authors:** Chuanjiang Li, Kai Wang, Linghong Guo, Hang Sun, Hai Huang, XinXin Lin, Qingping Li

**Affiliations:** 1 Department of Hepatobiliary Surgery, Nanfang Hospital, Southern Medical University, Guangzhou, Guangdong 510515, China; 2 Department of Surgery, Linzhi Municipal People's Hospital, Linzhi, Tibet 860100, China; 3 Department of Surgery, University of Pittsburgh Medical Center, Pittsburgh, PA 15261, USA; 4 The First Clinical College, Southern Medical University, Guangzhou, Guangdong 510515, China

**Keywords:** Hypoxia/Reoxygenation injury, MiR-34a-5p, Autophagy, Steatosis

## Abstract

Hypoxia-reoxygenation (H/R) injury in steatotic hepatocytes has been implicated in liver dysfunction after liver transplantation. MicroRNAs (miRs) play important roles in regulating several cell biology mechanisms related to H/R injury. However, the role of miRs in regulating H/R injury in steatotic hepatocytes is still unclear. We established an *in vitro* model for studying H/R injury in steatotic hepatocytes and identified miR-34a-5p as a miR that was substantially upregulated in steatotic hepatocytes under H/R challenge. MiR-34a-5p expression was modified by transfecting miR-34a-5p mimic and inhibitor into H/R-challenged steatotic hepatocytes. We found that inhibition of miR-34a-5p alleviated H/R-induced apoptosis and promoted post-H/R proliferation in steatotic hepatocytes. Whereas, overexpression of miR-34a-5p augmented H/R-induced apoptosis and prohibited post-H/R proliferation. By examining autophagy, our data demonstrated that miR-34a-5p suppressed autophagy in H/R-challenged steatotic hepatocytes, induction of autophagy partially rescued the exaggeration of H/R injury induced by miR-34a-5p mimic, while inhibition of autophagy impaired the protection of the miR-34a-5p inhibitor against H/R injury. In conclusion, miR-34a-5p is crucial in exaggerating H/R injury, likely by suppressing autophagy in steatotic hepatocytes. Inhibition of miR-34a may be a promising strategy to protect steatotic hepatocytes against H/R-injury.

## Abstract (precis)

**Summary:** Our study establishes an *in vitro* model for studying H/R injury in fatty liver, and shows that MiR-34a plays an important role in worsening H/R injury, likely by suppressing autophagy in steatotic hepatocytes.

## INTRODUCTION

Fatty liver disease has become the most common chronic liver disease in developed countries. Meanwhile, its prevalence is increasing rapidly in developing countries (Vernon et al., 2011). The shortage of donor livers for liver transplantation makes mild-to-moderate fatty liver grafts the major source of marginal liver donors (Attia et al., 2008; Pais et al., 2016). Hypoxia-reoxygenation (H/R) injury that occurs during ischemia-reperfusion injury has been implicated in liver transplantation and its severity correlates with liver dysfunction after surgery (Saikumar et al., 1998; Li and Jackson, 2002). H/R challenge exaggerates hypoxic injury after reoxygenation by augmenting oxidative stress, thus worsening tissue damage. Previous studies have shown that steatosis sensitizes hepatocyte to H/R injury (Berthiaume et al., 2009; Nativ et al., 2014). Although increased susceptibility of steatosis to H/R injury is thought to be associated with lipid accumulation (Berthiaume et al., 2009; Nativ et al., 2014), the precise mechanism has not yet been fully elucidated.

MicroRNAs (miRs) function in RNA silencing and post-transcriptional regulation of gene expression, which thus affects pathophysiological processes (Vidigal and Ventura, 2015), including H/R injury (Liu et al., 2014; Zhang et al., 2014; Wang et al., 2016; Huang et al., 2017). MiRs have various functions in regulating H/R injury through different mechanisms (Liu et al., 2014; Zhang et al., 2014; Wang et al., 2016; Huang et al., 2017). MiR-142-3p and miR-21 were reported to protect cells from H/R injury (Wang et al., 2016; Huang et al., 2017), while miR-15b, miR-133a-5p, and miR-92a were shown to deteriorate H/R injury (Liu et al., 2014; Zhang et al., 2014; Hao et al., 2017). MiR-34a is a multifunctional regulator of cell death and cell survival that is associated with H/R injury (Tazawa et al., 2007; Hermeking, 2010), and has been reported to induce apoptosis and cell-cycle arrest by targeting p53 in cancer cells (Hermeking, 2010). However, whether miR-34a regulates H/R injury in steatotic hepatocytes remains unknown.

Autophagy is a fine-tuning mechanism against cell injury that disassembles unnecessary or dysfunctional components to maintain cell homeostasis (Glick et al., 2010). There is emerging evidence showing that autophagy is also involved in H/R injury (Fan et al., 2017; Huang et al., 2017). In an *in vitro* model of H/R injury of alveolar epithelial cells, activation of autophagy by rapamycin pre-treatment was shown to protect the cells from H/R injury (Fan et al., 2017). The protective role of autophagy against H/R injury was also found in cardiac cells (Wu et al., 2015; Liu et al., 2017). Recently, miRs were found to regulate autophagy in H/R injury (Wu et al., 2015; Huang et al., 2017; Liu et al., 2017). Inhibition of miR-130a and miR-101 were shown to enhance H/R-induced autophagy in cardiac cells by regulating ATG14 and RAB5A, respectively (Wu et al., 2015; Liu et al., 2017). Huang et al. showed that miR-21 inhibited H/R-induced autophagy likely by regulating Akt/mTOR signaling pathway in cardiac cells (Huang et al., 2017). These studies together indicate that miRs play diverse roles in regulating H/R-induced autophagy in cardiac cells; however, how miRs affect H/R injury by altering autophagy in steatotic hepatocytes has not yet been investigated.

In this study, we establish an *in vitro* cell model to investigate the mechanism of H/R injury in steatotic hepatocytes. Our investigation reveals that miR-34a is substantially upregulated in steatotic hepatocytes under H/R conditions, and its upregulation exaggerates cell apoptosis and prohibits cell proliferation, likely by suppressing protective autophagy program. Our findings thus suggest that miR-34a may be a therapeutic target for H/R injury in steatotic hepatocytes.

## RESULTS

### Establish an *in vitro* model for studying H/R injury in fatty liver

**Fig. 1.**
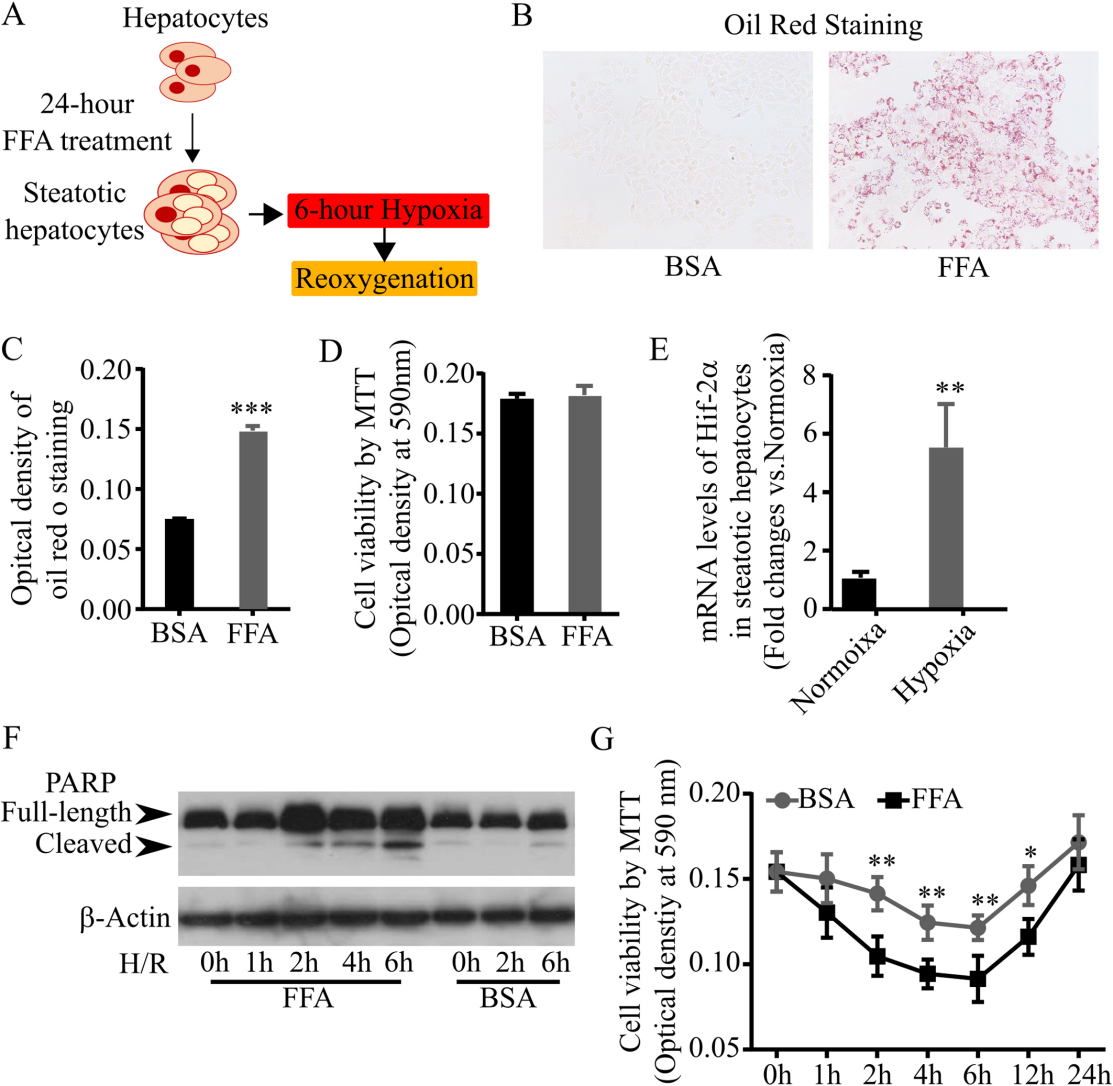
**Establishment of an *in vitro* model for studying H/R injury in fatty liver.** (A) Experimental design. L02 cells were fed with FFA to induce steatosis then challenged with hypoxic condition (0.1% oxygen) for 6 h followed by reoxygenation for up to 24 h. (B,C) Steatosis in L02 cells. Oil Red O-stained FFA-treated and BSA-treated L02 cells were observed under microscopy (B) and optical density was measured (C). (D) Cell viability. Cell viability was measured by MTT assay after 24 h induction of steatosis. (E) mRNA levels of Hif-2α in hypoxia-challenged steatotic hepatocyte. Total mRNA were extracted from FFA-treated L02 cells after 6 h hypoxic culture or normoxic culture, then subjected real-time PCR to measure Hif-2α mRNA levels. (F) Apoptosis in H/R-challenged steatotic hepatocytes. Proteins were extracted from FFA-treated and BSA-treated L02 cells after H/R challenge, then subjected to immunoblotting to detect PARP and its cleaved form. (G) Cell viability of H/R-challenged steatotic hepatocytes. MTT assay was performed to measure cell viability of FFA-treated and BSA-treated L02 cells after H/R challenge. Data represent three independent experiments. ***P<0.001; **P<0.05.

H/R injury is a key factor in liver dysfunction after liver transplantation (Zhai et al., 2013). To investigate how H/R stimulation damages steatotic hepatocytes, we first established an *in vitro* model of H/R-injured fatty liver. L02 cell is an immortalized human hepatocyte cell line which has been widely used to study various physiopathologies of liver diseases (Leung et al., 2011; Xiang et al., 2011). In our study, L02 cells were fed with a free fatty acid (FFA) mixture of palmitic acid and oleic acid for 24 h to induce steatosis (Fig. 1A). By staining FFA-treated L02 cells with Oil Red O, we found that FFA treatment significantly enhanced the staining (Fig. 1B,C), indicating that FFA treatment indeed induced lipid accumulation in L02 cells. Furthermore, by measuring cell viability with a MTT assay, we found that the treatment of the FFA mixture did not significantly reduce cell viability compared to BSA treatment (Fig. 1D).

Next, to induce H/R injury, steatotic L02 cells were cultured in hypoxic conditions for 6 h followed by reoxygenation for up to 24 h (Fig. 1A). Real-time PCR assay revealed a significant upregulation of hypoxia marker HIF-2α in steatotic L02 cells after hypoxic culture (Fig. 1E), suggesting that steatotic L02 cells were suffering from hypoxia. It is reported that H/R injury reduces cell viability, likely by inducing apoptosis (Saikumar et al., 1998; Li and Jackson, 2002), and steatosis sensitizes hepatocytes to H/R-induced apoptosis (Berthiaume et al., 2009; Nativ et al., 2014). To examine cellular injury in H/R-challenged steatotic L02 cells, apoptosis was measured. By immunoblotting an apoptosis marker PARP, our results showed cleaved PARP was elevated to a higher level in steatotic L02 cells than in BSA-treated L02 cells in the first 6 h after reoxygenation (Fig. 1F), suggesting that H/R stimulation indeed induced apoptosis in L02 cells, and steatosis worsened the apoptosis. Hepatocytes have the potential to proliferate in response to liver injury (Michalopoulos, 2017). By MTT assay, we found that viability of L02 cells decreased in the first 6 h, and started to recover at 12 h after reoxygenation (Fig. 1G). Steatosis significantly accelerated H/R-induced cell death and delayed the recovery after H/R challenge (Fig. 1G), indicating that steatosis weakens the potential to overcome H/R injury in hepatocytes. Altogether, these data demonstrate that treating L02 cells with FFA to induce steatosis, then challenging the steatotic L02 cells with H/R condition to induce H/R injury, can provide a convenient *in vitro* model for studying H/R injury in steatotic hepatocytes.

### MiR-34a-5p expression is substantially unregulated in H/R-challenged steatotic hepatocytes

**Fig. 2.**
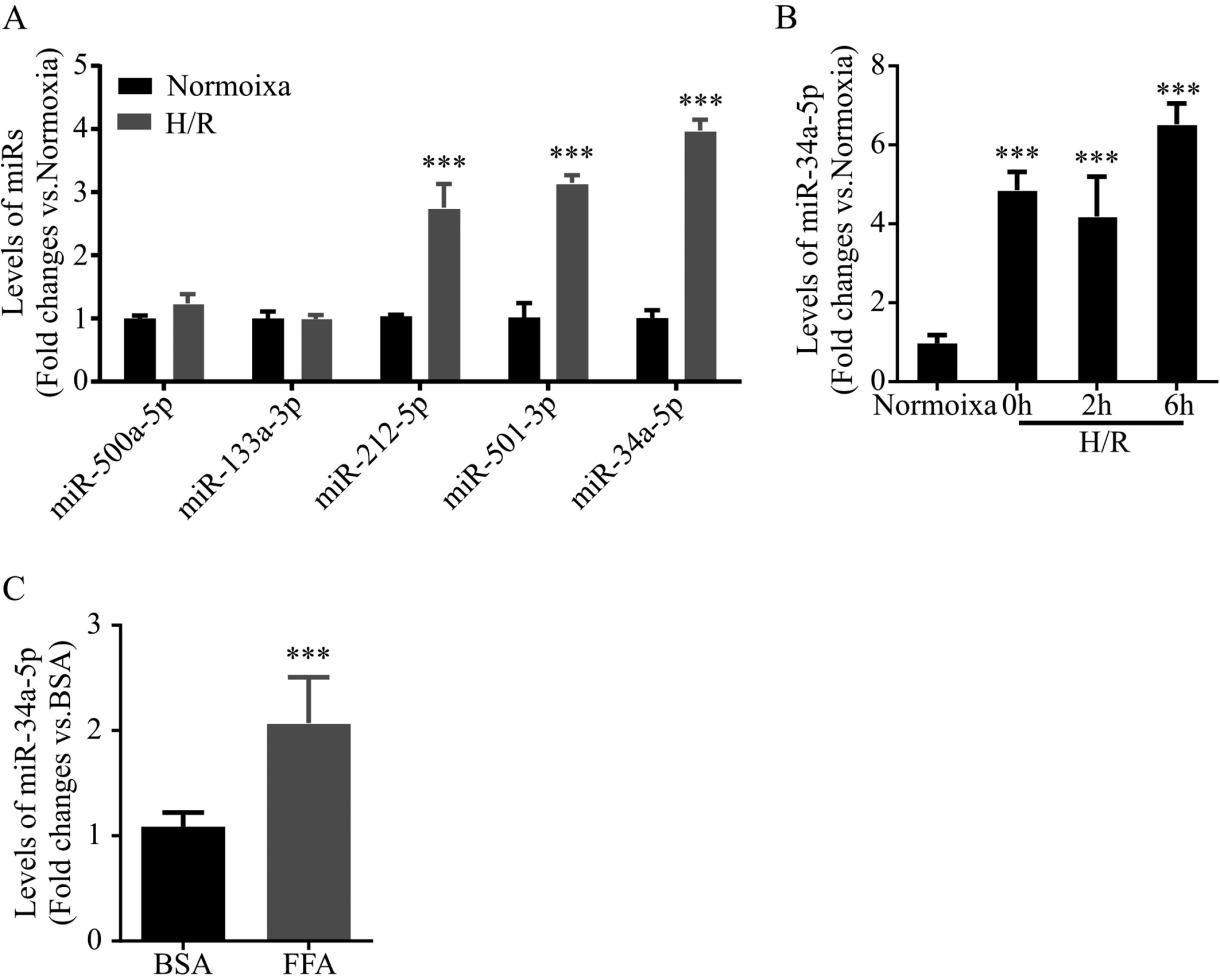
**MiR-34a-5p expression is substantially unregulated in H/R-challenged steatotic hepatocytes.** (A) Levels of miRs in H/R-challenged steatotic hepatocyte. Total miRs were extracted from FFA-treated L02 cells after 6 h hypoxic culture followed by 2 h reoxygenation, then levels of miR-550a-5p, miR-133a-3p, miR-212-5p, miR-501-3p, and miR-34a-5p were measured by real-time PCR. (B) Levels of miR-34a-5p in H/R-challenged steatotic hepatocyte. Total miRs were extracted from FFA-treated L02 cells after 6 h hypoxic culture followed by reoxygenation at indicated time points, then levels of miR-34a-5p were measured. (C) Levels of miR-34a-5p in H/R-challenged steatotic hepatocyte and normal hepatocytes. Levels of miR-34a-5p were measured in BAS-treated and FFA-treated L02 cells after 6 h hypoxic culture followed by 6 h reoxygenation. Data represent three independent experiments. ***P<0.001.

H/R injury is an essential mechanism of ischemia-reperfusion injury (Saikumar et al., 1998). Several miRs have been shown as biological signatures of ischemia-reperfusion injury, including miR-500, miR-133a, miR-212, miR-34a, and miR-501 (Godwin et al., 2010). To investigate whether these miRs regulated H/R injury in steatotic hepatocytes, miR levels were measured by real-time PCR assay in steatotic L02 cells that were cultured in hypoxic conditions for 6 h followed by 2 h reoxygenation. The data showed that miR-212-5p, miR-34a-5p, and miR-501-3p were significantly upregulated in H/R-challenged steatotic L02 cells (Fig. 2A). Among these miRs, miR-34a-5p had the greatest upregulation (Fig. 2A). MiR-34a regulates diverse cell biology associated with H/R injury (Tazawa et al., 2007; Hermeking, 2010). Therefore, to further illustrate the dysregulation of miR-34a-5p in H/R-challenged hepatocytes, we determined levels of miR-34a-5p in H/R-challenged steatotic L02 cells at different time points after reoxygenation. We found a persistent upregulation of miR-34a-5p in steatotic L02 cells during the first 6 h after reoxygenation (Fig. 2B). Furthermore, compared to normal hepatocytes, miR-34a-5p was increased to a higher level in steatotic hepatocytes at 6 h after reoxygenation (Fig. 2C). These data suggest that miR-34a-5p is substantially upregulated in H/R-challenged steatotic hepatocytes.

### MiR-34a-5p augments H/R-induced apoptosis in steatotic hepatocytes

**Fig. 3.**
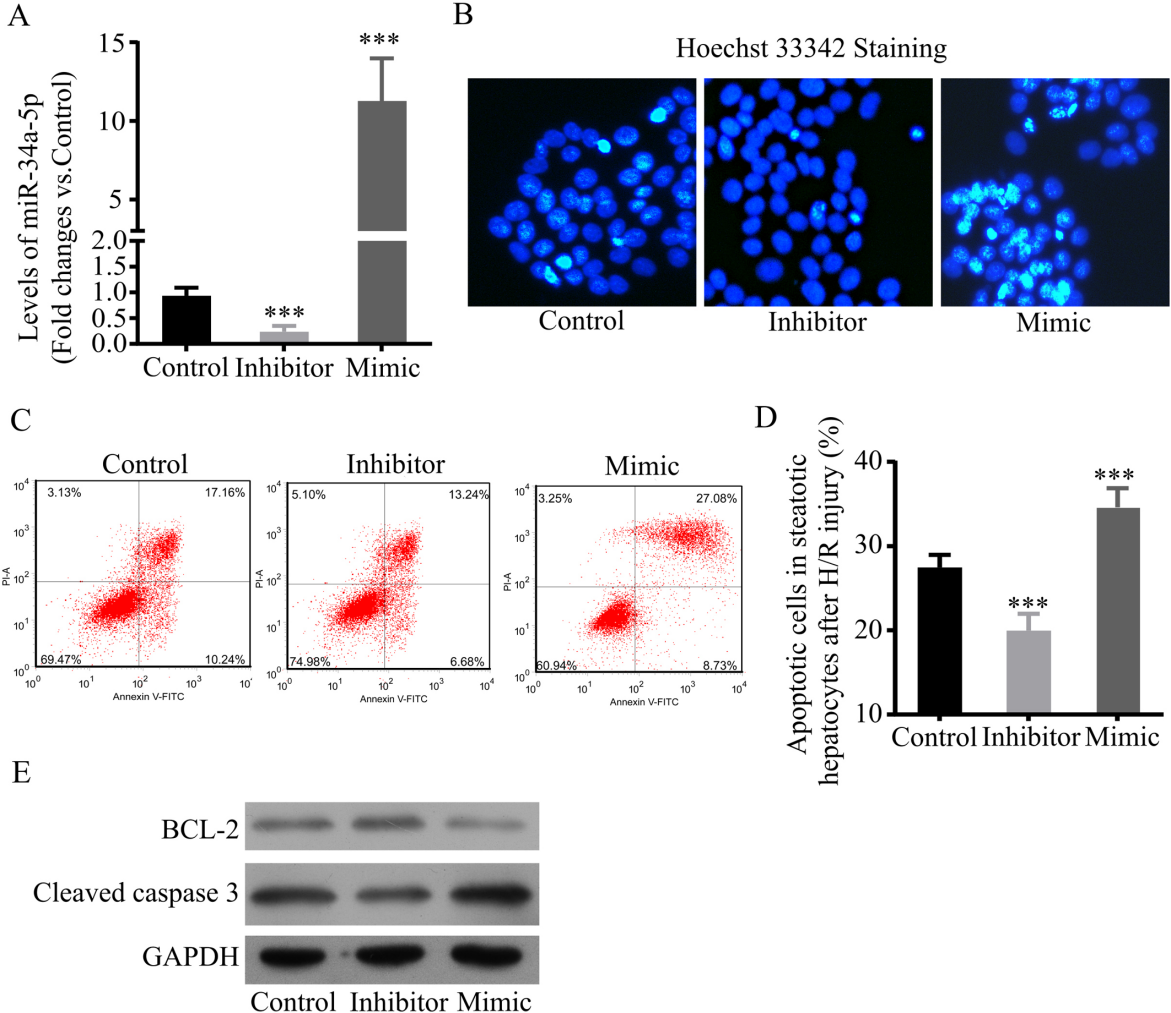
**MiR-34a-5p augments H/R-induced apoptosis in steatotic hepatocytes.** (A) Levels of miR-34a-5p in steatotic hepatocyte after transfection. At 24 h after transfection, total miRs were extracted from miR-34a-5p inhibitor, mimic, and negative control miR-transfected steatotic L02 cells, then levels of miR-34a-5p were measured by real-time PCR. (B-E) Apoptosis in transfected steatotic L02 cells under H/R challenge. Transfected steatotic L02 cells were challenged with 6 h hypoxia followed by 6 h reoxygenation. Cells were fixed and subjected to Hoechst 33342 staining (B), quantification of PI and Annexin V-stained cells by FACS (C,D), and immunoblotting of BCL-2 and cleaved caspase-3 (E) to detect apoptosis. Data represent three independent experiments. ***P<0.001.

MiR-34a has been proved to be a multifunctional regulator in cell division, senescence, and apoptosis (Tazawa et al., 2007; Hermeking, 2010). Since we observed a substantial upregulation of miR-34a-5p in steatotic L02 cells under H/R challenge, we hypothesized that miR-34a-5p might play an important role in regulating H/R injury in steatotic hepatocytes. To test this hypothesis, the steatotic L02 cells were first transfected with miR-34a-5p inhibitor, mimic, and negative control miR, respectively. Real-time PCR assay determined that miR-34a-5p inhibitor downregulated miR-34a-5p while the mimic upregulated miR-34a-5p, compared to negative control (Fig. 3A). Then the transfected cells were subjected to 6 h hypoxia followed by 6 h reoxygenation to induce H/R injury. To examine the influence of miR-34a-5p on H/R-induced apoptosis, we first performed Hoechst 33342 staining. We observed that miR-34a-5p inhibitor significantly reduced apoptosis, whereas miR-34a-5p mimic significantly increased apoptotic cells (Fig. 3B). In line with these results, by quantifying the apoptotic cells stained with PI and Annexin V using FACS, we also found that the number of apoptotic cells in miR-34a-5p inhibitor-transfected cells was significantly lower than in control cells (Fig. 3C,D). However, apoptotic cells in miR-34a-5p mimic-transfected cells were significantly increased compared to control cells (Fig. 3C,D). Finally, we performed immunoblotting to detect an apoptosis marker, cleaved caspase-3, and an anti-apoptotic marker, BCL-2. The result showed that miR-34a-5p inhibitor reduced expression of cleaved caspase-3 and slightly increased expression of BCL-2 in H/R challenged steatotic L02 cells, while miR-34a-5p mimic behaved in the opposite manner (Fig. 3E). These findings demonstrate that the inhibition of miR-34a-5p alleviates H/R-induced apoptosis, while elevation of miR-34a-5p worsens H/R-induced apoptosis, indicating that upregulation of miR-34a-5p in steatotic hepatocytes under H/R condition is important in promoting H/R-induced apoptosis.

### MiR-34a-5p prohibits proliferation in steatotic hepatocytes after H/R injury

**Fig. 4.**
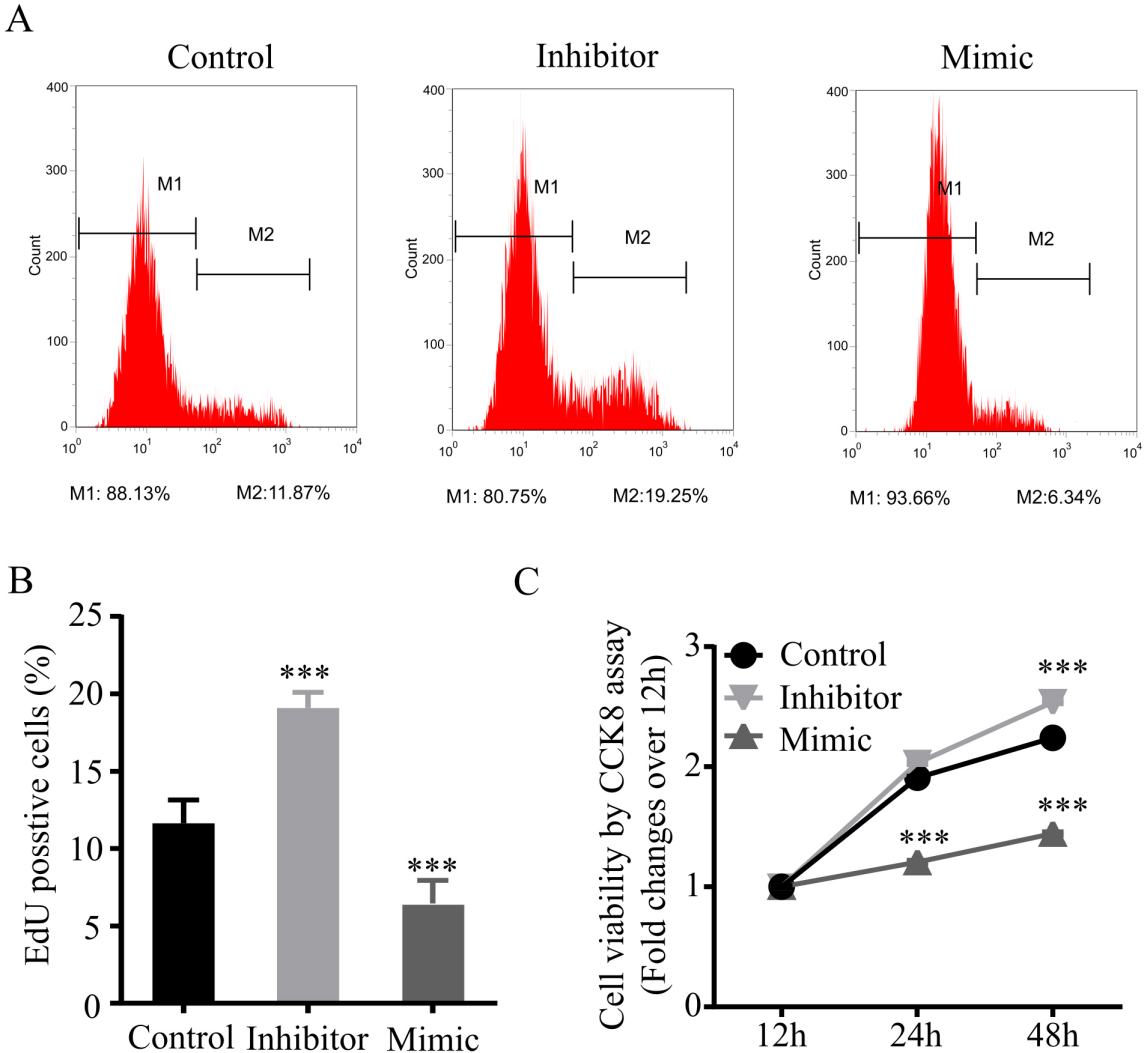
**MiR-34a-5p prohibits proliferation in steatotic hepatocytes after H/R injury.** (A,B) Edu labeling proliferation assays. MiR-34a-5p mimic, miR-34a-5p inhibitor, and negative control miR transfected steatotic L02 cells were subjected to 6 h hypoxia followed by reoxygenation for 48 h. Cells were labeled with EdU (A) and quantified by flow cytometry (B). (C) Time-course cell viability measured by CCK-8 assays. MiR-34a-5p mimic, miR-34a-5p inhibitor, and negative control miR transfected steatotic L02 cells were collected at indicated time points after reoxygenation, and cell viability was measure by subjected to CCK-8 assay. Data represent three independent experiments. ***P<0.001.

Hepatocytes have the potential to proliferate in response to liver injury (Michalopoulos, 2017). In our model, we also found that H/R-challenged steatotic L02 cells continued to proliferate after reoxygenation. Therefore, we determined whether miR-34a-5p played a role in regulating proliferation in steatotic hepatocyte after H/R challenge. We performed Edu labeling proliferation assays on miR-34a-5p mimic and inhibitor transfected steatotic L02 cells at 48 h after reoxygenation. As shown in Fig. 4A and B, compared to control cells, the percentage of EdU-positive cells in miR-34a-5p inhibitor-transfected cells was significantly increased, while it was significantly reduced by miR-34a-5p mimic transfection. A time-course study using CCK8 assay further revealed a lower viability in miR-34a-5p mimic-transfected cells, and a higher viability in miR-34a-5p inhibitor-transfected cells at both 24 h and 48 h after reoxygenation, compared to control cells (Fig. 4C). These findings show that, during the recovery from H/R injury, inhibition of miR-34a-5p promotes proliferation in steatotic hepatocytes, however, overexpression of miR-34a-5p prohibits the proliferation. Therefore, miR-34a-5p is likely a negative player of proliferation in steatotic hepatocytes after H/R injury.

### MiR-34a-5p suppresses autophagy in steatotic hepatocytes under H/R condition

**Fig. 5.**
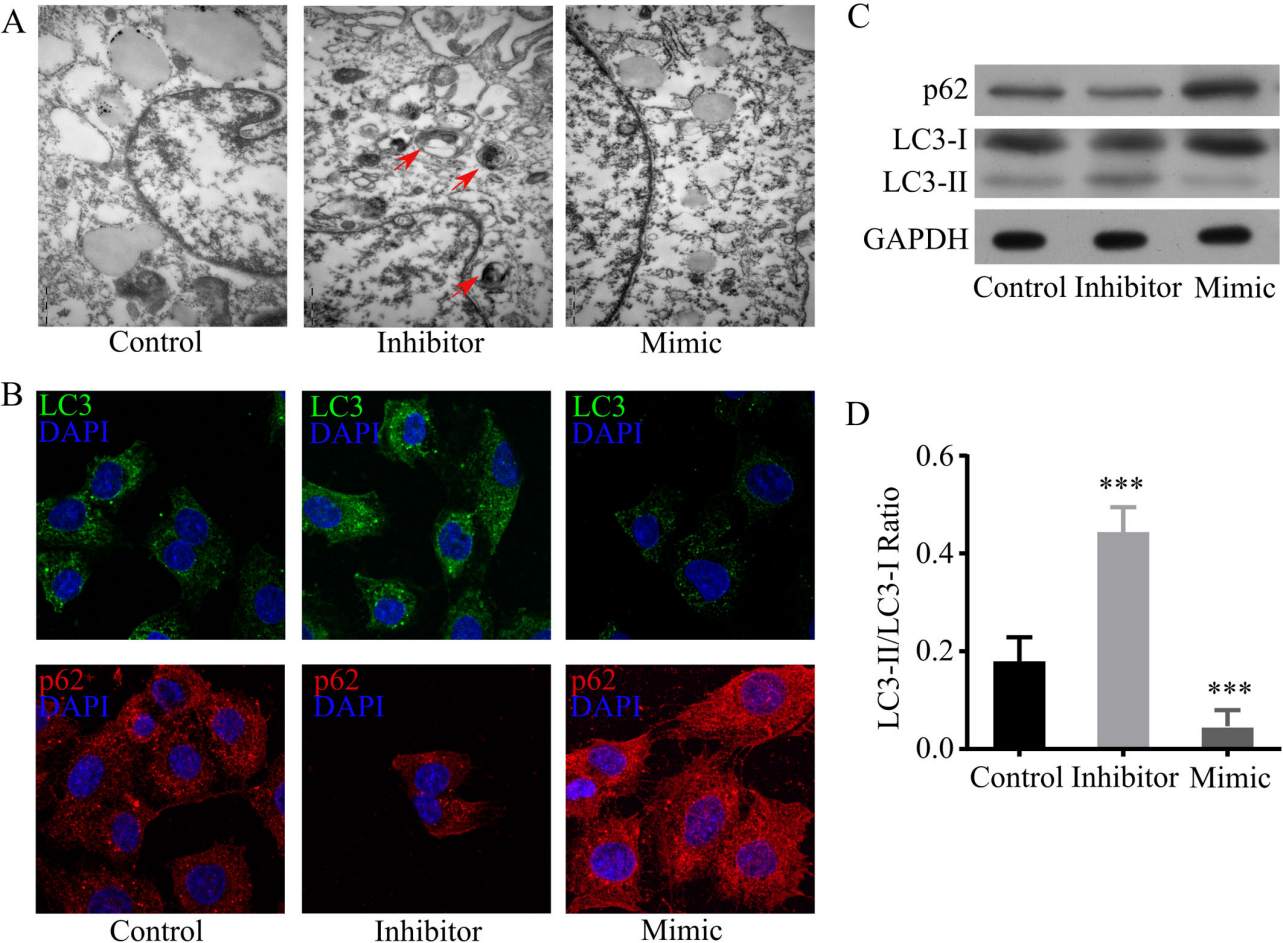
**MiR-34a-5p suppresses autophagy in steatotic hepatocytes under H/R condition.** (A) Electronic microscopic images of H/R-challenged steatotic hepatocytes. After miR-34a-5p mimic, miR-34a-5p inhibitor, and negative control mi-RNA transfection, steatotic L02 cells were subjected to 6 h hypoxia followed by 6 h reoxygenation. Cells were fixed for electronic microscopy scanning; red arrows indicate autophagosomes in cells. (B) Immunofluorescence staining of LC3 and p62 in H/R-challenged steatotic hepatocytes. Transfected steatotic L02 cells were subjected to H/R stimulation. Cells were fixed and stained with LC3 and p63 antibody followed with Alexa Fluor 488-conjugated and Rhodamine-conjugated secondary antibodies, respectively. Immunofluorescence staining was observed under fluorescence microscope. (C,D) Immunoblotting of LC3 and p62 in H/R-challenged steatotic hepatocytes. Proteins were extracted from transfected steatotic L02 cells after H/R challenge, then subjected to immunoblotting of LC3-I, LC3-II, and p62 (C). The ratio of LC3-II/LC3-I was quantified by measuring band intensity using ImageJ software (D). Data represent three independent experiments. ***P<0.001.

Autophagy is a critical process that affects both apoptosis and proliferation (Glick et al., 2010). MiR-34a has been recently shown to suppress autophagy (Yang et al., 2013; Huang et al., 2014). Since our results demonstrated that miR-34a-5p regulated both apoptosis and proliferation in H/R-challenged steatotic hepatocytes, we proposed that miR-34a-5p might regulate autophagy in steatotic hepatocytes during H/R injury. To examine this hypothesis, after miR-34a-5p mimic and inhibitor transfection, steatotic L02 cells were subjected to 6 h hypoxia followed by 6 h reoxygenation to induce apoptosis. We first observed autophagosomes in transfected steatotic hepatocytes by electronic microscopy. The results displayed a higher amount of autophagosomes in miR-34a-5p inhibitor-transfected steatotic hepatocytes than mimic-transfected and control cells (Fig. 5A), indicating that miR-34a-5p inhibitor enhanced autophagy in H/R-challenged steatotic hepatocytes. We next measured the expression of autophagy-associated proteins. LC3 is a protein positively correlated with induction of autophagy, and p62 is a protein negatively correlated with autophagy, both of them were immunostained and immunoblotted. We found that miR-34a-5p inhibitor upregulated LC3 and decreased p62. However, miR-34a-5p mimic downregulated LC3 and increased p62 (Figs 5B and 3C). Conversion of LC3-I to LC3-II is a hallmark of autophagy in mammalian cells. By immunoblotting both LC3-1 and LC3-II, we found that the ratio of LC3-II/LC3-I was significantly elevated by miR-34a-5p inhibitor transfection, and it was significantly reduced by miR-34a-5p mimic transfection (Fig. 5C,D), indicating a high conversion and a low conversion of LC3-I to LC3-II in inhibitor-transfected cells and mimic-transfected cells, respectively. These findings show that inhibition of miR-34a-5p enhances autophagy, while overexpression of miR-34a-5p suppresses autophagy in H/R-challenged steatotic hepatocyte, suggesting that miR-34a-5p is a potent inhibitor of autophagy in steatotic hepatocytes under H/R condition.

### MiR-34a-5p regulates H/R injury in steatotic hepatocytes by altering autophagy

**Fig. 6.**
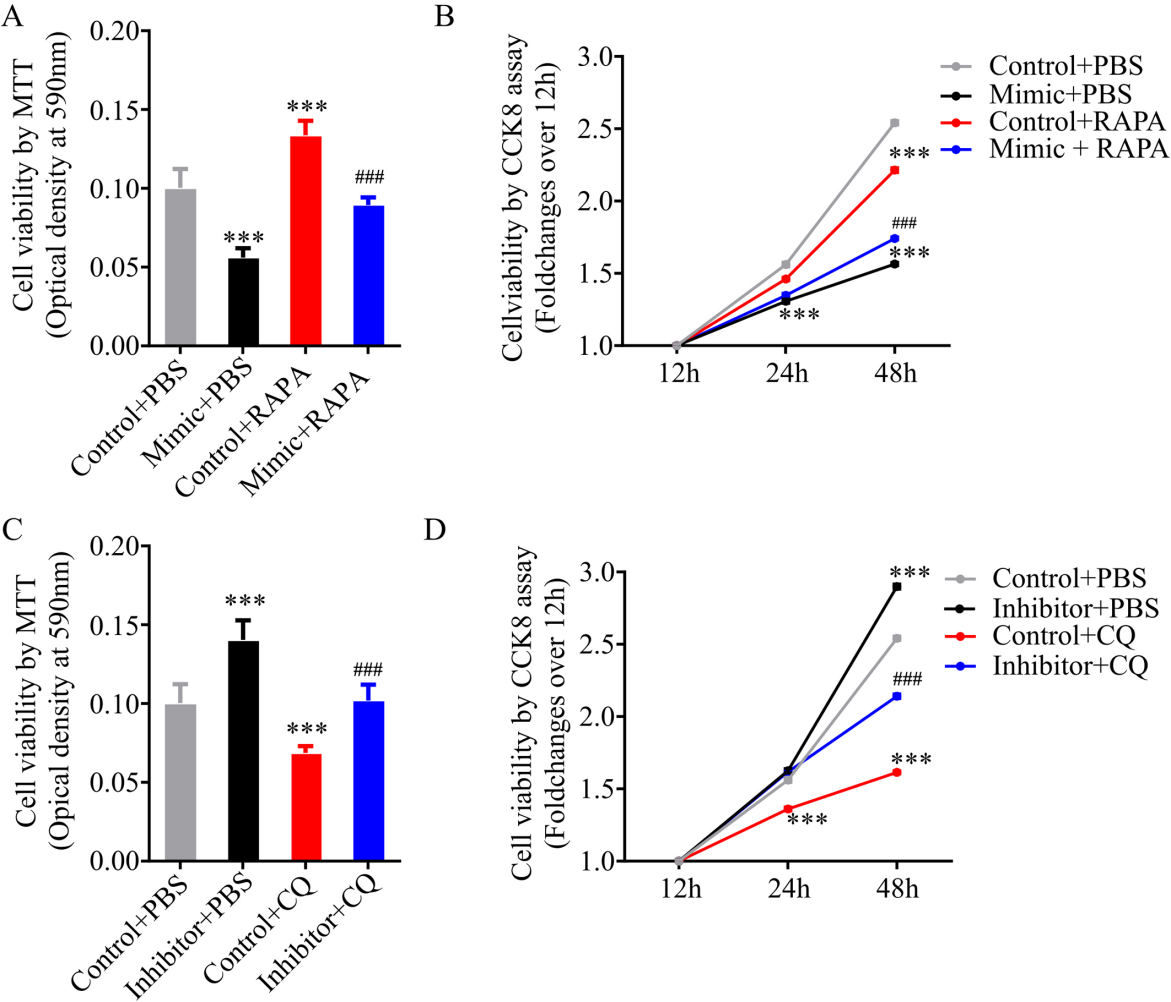
**MiR-34a-5p regulates H/R injury in steatotic hepatocytes by altering autophagy.** (A,B) 5 µM rapamycin (RAPA) was added to cell media to induce autophagy during H/R challenge. MTT assay was performed to measure cell viability of steatotic hepatocytes after 6 h hypoxic culture followed by 6 h reoxygenation (A). Time-course cell viability measured by CCK-8 assays (B). (C,D) 10 µM chloroquine (QC) was added to cell media to inhibit autophagy during H/R challenge. MTT assay was performed to measure cell viability of steatotic hepatocytes after 6 h hypoxic culture followed by 6 h reoxygenation (C). Time-course cell viability measured by CCK-8 assays (D). Data represent three independent experiments. ***P<0.001, versus Control+PBS; ^###^*P*<0.001, versus Mimic+PBS or Inhibitor+PBS.

Autophagy has been shown to be a protective program against cell injury (Liu et al., 2014; Zhang et al., 2014; Wang et al., 2016; Huang et al., 2017). Several studies have demonstrated that induction of autophagy mediated by miRs, protects cells against H/R injury (Liu et al., 2014; Zhang et al., 2014; Wang et al., 2016; Huang et al., 2017). Therefore, we postulated that the upregulation of miR-34a-5p exaggerated H/R injury in steatotic L02 cells was caused by suppressing autophagy. To test this hypothesis, we first investigated if induction of autophagy reduced the exaggeration of H/R injury mediated by miR-34a-5p mimic. Rapamycin was used as an autophagic inducer during H/R challenge. We found that rapamycin alleviated H/R-induced cell death after 6 h hypoxia followed by 6 h reoxygenation (Fig. 6A); however, it failed to promote cell proliferation after H/R injury (Fig. 6B). In miR-34a-5p mimic-transfected steatotic hepatocytes, rapamycin treatment significantly alleviated cell death and promoted cell proliferation upon H/R challenge (Fig. 6A,B). These data suggest that the exaggeration of H/R injury mediated by miR-34a-5p mimic can be partially reduced by rapamycin treatment. We then studied if inhibition of autophagy abolished the protective effects of miR-34a-5p inhibitor against H/R injury. Chloroquine was used as an autophagic inhibitor during H/R challenge. We found that chloroquine worsened H/R-induced cell death after 6 h hypoxia followed by 6 h reoxygenation (Fig. 6C). During recovery phase after H/R injury, chloroquine prohibited cell proliferation (Fig. 6D). In miR-34a-5p inhibitor-transfected steatotic hepatocytes, chloroquine treatment significantly increased cell death and suppressed cell proliferation upon H/R challenge (Fig. 6C,D). These data suggest that the protection against H/R injury by miR-34a-5p inhibitor is weakened by chloroquine treatment. In conclusion, these data indicate that autophagy is a potent mechanism by which miR-34a-5p regulates H/R injury in steatotic hepatocytes.

## DISCUSSION

Currently, the increasing incidence of fatty liver diseases makes fatty liver an important donor for liver transplantation, but fatty liver grafts are more likely to cause abnormal liver function and severe complications than normal liver grafts (Attia et al., 2008; Pais et al., 2016). H/R injury is a key factor that mediates cell death and cell survival in liver grafts suffering from ischemia and reperfusion (Zhai et al., 2013). However, how H/R injury affects steatotic grafts is not yet clear. To investigate this, we established an *in vitro* model for studying H/R injury in fatty liver. We found that H/R challenge indeed induced apoptosis in steatotic hepatocytes, thereafter, steatotic hepatocytes were able to recover from H/R injury and continued to proliferate. Next, we detected that miR-34a was greatly upregulated in H/R-challenged steatotic hepatocytes. Moreover, we found that inhibition of miR-34a-5p alleviated H/R-induced apoptosis and promoted post-H/R proliferation in steatotic hepatocytes, whereas overexpression of miR-34a-5p augmented H/R-induced apoptosis and prohibited post-H/R proliferation. Mechanically, we found that the exaggerated H/R injury induced by miR-34a was likely associated with autophagy inhibition. Our findings thus suggest that miR-34a is a potential target for protecting fatty liver grafts against H/R injury.

*In vitro* cell models of steatosis provide a convenient way to study fatty liver diseases (Gomez-Lechon et al., 2007; Ricchi et al., 2009). Palmitic acid and oleic acid are the most common fatty acids used in cell culture to induce steatosis in hepatocytes (Gomez-Lechon et al., 2007). The L02 cell line is an immortalized human hepatocyte cell line that is widely used as an *in vitro* model for liver diseases (Leung et al., 2011; Xiang et al., 2011). Here, we established an *in vitro* fatty model by feeding L02 cells with a mixture of oleic acid and palmitic acid. We confirmed that neutral lipids were indeed accumulated in L02 cells, while cell survival was not significantly reduced. Although palmitic acid has been shown to cause cell death in hepatocytes, oleic acid treatment alleviates palmitic-acid-induced cell death (Listenberger et al., 2003; Ricchi et al., 2009). This is likely because lipogenesis, enhanced by oleic acid, drains free palmitic acid into the synthesis of neutral lipids, thus reducing the level of free palmitic acid in cells (Listenberger et al., 2003; Ricchi et al., 2009). Therefore, the mixture of oleic acid and palmitic acid did not induce obvious cell death in our model. In addition, aiming to mimic H/R condition in fatty grafts of liver transplantation, we challenged the steatotic L02 cells with 6 h hypoxic culture followed by reoxygenation. H/R challenge has been shown to induce apoptosis in different cells (Li and Jackson, 2002). Similar to this, we also observed that H/R challenge induced apoptosis in the steatotic L02 cells. Interestingly, H/R injury was found to last about 6 h after reoxygenation in our model, by then the steatotic L02 cells started to recover and continued to proliferate. This suggests that, like normal hepatocytes, which can proliferate to overcome liver injury, the steatotic L02 cells also have the potential to recover from H/R injury. Altogether, these findings indicate that our model provides a convenient way to study hepatocellular injury and recovery under H/R challenge.

There is emerging evidence showing that miRs play regulatory roles in H/R injury (Liu et al., 2014; Zhang et al., 2014; Wang et al., 2016; Huang et al., 2017). In this study, we first found that miR-34a was greatly and persistently elevated in H/R-challenged steatotic L02 cells. The miR-34a family has been reported to induce apoptosis and growth arrest in cancer cells (Hermeking, 2010). In line with this, we showed that miR-34a-5p exaggerated apoptosis and prohibited proliferation in H/R-challenged steatotic L02 cells. We conclude that the upregulation of miR-34a-5p is important to promote H/R injury in steatotic L02 cells. Mitochondrial dysfunction as a consequence of oxidative stress plays a central role in H/R injury (Weinberg et al., 2000). When mitochondrial dysfunction occurs, a decrease of BCL-2 elevates pro-apoptotic proteins to induce apoptosis (Susin and Zamzami, 1996). MiR-34a was shown to mediate palmitate-induced apoptosis by downregulating BCL-2 expression (Lin et al., 2014). We also observed a downregulation of BCL-2 in miR-34a-5p-overexpressing steatotic L02 cells under H/R challenge. Therefore, we postulate that miR-34a augments H/R injury in steatotic L02 cells, likely by worsening mitochondrial dysfunction. In H/R-challenged steatotic L02 cells, miR-212 and miR-501 were also significantly upregulated, but to a lesser extent than miR-34a. Although the elevations of miR-212 and miR-501 were previously reported as potential biological causes of ischemia-reperfusion injury (Godwin et al., 2010), their function in regulating H/R injury requires more investigation.

MiR-34a has been recently shown to suppress autophagy (Yang et al., 2013; Huang et al., 2014). A previous study from Yang's group showed that miR-34 modulated *Caenorhabditis elegans* lifespan by repressing autophagy (Yang et al., 2013). Our presented data revealed that miR-34a-5p overexpression reduced autophagosomes, and downregulated p62 and LC3 expression, indicating a negative role of miR-34a-5p in autophagy in H/R-challenged steatotic L02 cells. Although the mechanism by which miR-34a-5p suppressed autophagy in H/R-challenged steatotic hepatocytes was not elucidated in our study, a study by Huang et al. demonstrated that miR-34a suppressed autophagy by inhibiting ATG9A expression (Huang et al., 2014); we considered that this might also occur in H/R-challenged steatotic hepatocytes.

Autophagy is known to be a protective program against cell injury (Liu et al., 2014; Zhang et al., 2014; Wang et al., 2016; Huang et al., 2017). Several studies have shown that induction of autophagy mediated by miRs protects cells against H/R injury (Liu et al., 2014; Zhang et al., 2014; Wang et al., 2016; Huang et al., 2017). In our study, we found that induction of autophagy by rapamycin treatment alleviated H/R-induced cell death in steatotic hepatocytes, and partially reduced the miR-34a-5p-induced exaggeration of H/R injury. Meanwhile, inhibition of autophagy by chloroquine aggravated H/R injury in steatotic hepatocytes, and weakened the protection against H/R injury provided by the miR-34a-5p inhibitor. Therefore, we propose that the upregulation of miR-34a-5p exaggerates H/R injury in steatotic L02 cells and is partially caused by suppressing autophagy.

Taken altogether, our presented findings suggest that miR-34a plays an important role in worsening H/R injury likely by suppressing autophagy in steatotic hepatocytes. Inhibition of miR-34a may be a promising way to prevent H/R injury in steatotic hepatocytes.

## MATERIALS AND METHODS

### Establishment of *in vitro* H/R-challenged fatty liver model

The immortalized human hepatocyte L02 cell line is widely used as an *in vitro* model for several liver diseases (Leung et al., 2011; Xiang et al., 2011). The *in vitro* fatty liver model on L02 cells was established according to previous studies, with minor modifications (Gomez-Lechon et al., 2007). L02 cells were grown in 1640 medium supplemented with 10% fetal bovine serum under 5% CO_2_ and 95% humidity at 37°C. After seeding cells for 24 h, the control group was treated with serum-free 1640 medium containing 1% fatty-acid-free bovine serum albumin (BSA), while the fatty liver model group was treated with BSA-conjugated oleic acid and palmitic acid (Sigma-Aldrich) with the ratio at 1:1 (100 µM:100 µM). After another 24 h incubation, cells were washed with PBS three times and fixed by 4% paraformaldehyde. The lipid droplets were stained with Oil Red O (Sigma-Aldrich) and observed under microscope. Optical density was measured by a plate reader after eluting the dye using isopropanol. To induce H/R-injury, steatotic cells were subjected to hypoxia culture (0.1% oxygen) for 6 h followed by reoxygenation for indicated periods.

### RNA extraction and quantitative polymerase chain reaction (real-time PCR)

H/R-challenged steatotic cells were collected, and total RNA was isolated using RNeasy Mini Kit (Qiagen). The quantification of miR-550a-5p, miR-133a-3p, miR-212-5p, miR-501-3p, and miR-34a-5p was determined by quantitative real-time PCR. It was carried out using SuperScriptIII Platinum SYBR Green One-Step qRT-PCR kit (Invitrogen) by an ABI PRISM 7900 system (Applied Biosystems, Foster City, USA). The relative mRNA amount of miR-34a-5p and Hif-2α were calculated by comparative Ct method after normalizing against the quantity of RNU6 and β-Actin, respectively. The primer sequences were used as follows: miR-34a-5p: forward: 5′-AGGGGGTGGCAGTGTCTTAG-3′, reverse: 5′-GTGCGTGTCGTGGAGTCG-3′; RNU6: forward: 5′-GCTTCGGCAGCACATATACTAAAAT-3′, reverse: 5′-CGCTTCACGAATTTGCGTGTCAT-3′. HIF-2α: forward: 5′-TCGTCAGCCCACAAGGTGTC-3′, reverse: 5′-GGCACGTTCACCTCACAGTCA-3′. β-actin: forward: 5′-GATGTATGAAGGCTTTGGTC-3′, reverse: 5′-TGTGCACTTTTATTGGTCTC-3′.

### Transfection of miR-34a-5p mimic and inhibitor

MiR-34a-5p mimic, inhibitor and negative control miR were supplied by Ribobio Co. Ltd (Guangzhou, China). The steatotic L02 cells were transfected with miR-34a-5p mimic, inhibitor, and negative control miR using Lipofectamine 2000 (Invitrogen). After another 24 h incubation, total RNA was isolated from transfected cells, and quantification of miR-34a-5p was determined by quantitative real-time PCR. The sequences of miR-34a-5p inhibitor, mimic, and negative control were used as follows: miR-34a-5p inhibitor: 5′-ACAACCAGCUAAGACACUGCCA-3′, mimic: 5′-UGGCAGUGUCUUAGCUGGUUGU-3′; negative control: 5′-UUCUCCGAACGUGUCACGUTT-3′. To induce autophagy, 5 µM rapamycin was added to cell media during H/R challenge. To inhibit autophagy, 10 µM chloroquine was added to cell media during H/R challenge.

### Measurement of cell apoptosis

Assessment of apoptosis was carried out using flow cytometry analysis and Hoechst staining assay. For flow cytometry analysis, after H/R challenge, the transfected steatotic L02 cells were harvested and subjected to Annexin V and PI staining using an Annexin V-FITC apoptosis detection kit (BD Biosciences). Samples were then analyzed in a FACS system (BD Biosciences) and data were analyzed using Cellquest software (BD Biosciences). For Hoechst staining assay, cells were incubating with diluted Hoechst 33342 (Sigma-Aldrich) for 10 min. After washing with PBS, Hoechst-positive nuclei were observed by fluorescence microscopy (Leica, Wetzlar, Germany).

### Western blotting

Cells were lysed using protein extraction reagent supplied with protease inhibitor cocktail (Thermo Fisher Scientific). Total cellular proteins were extracted and the protein concentrations were determined by BCA Protein Assay Kit (Thermo Fisher Scientific). Equal amounts of protein lysates were separated by 10% SDS-PAGE and then transferred to PVDF membrane (Millipore). After blocking with 5% milk, the membranes were incubated with antibodies against cleaved casapase-3, BCL-2, LC3, p62, β-Actin and GAPDH (Cell Signaling Technology), respectively. HRP-conjugated secondary antibodies (Cell Signaling Technology) were applied and the signals were visualized using the ECL detection kit (GE Healthcare). Band intensity was measured using ImageJ software (NIH).

### Immunofluorescence staining

To investigate the effect of miR-34a-5p on autophagy activity, immunofluorescence staining was performed to evaluate the expression and distribution of LC3 and p62 protein. Cells were fixed with 4% paraformaldehyde and permeabilized in 0.5% Triton X-100 in PBS. After blocking with 3% BSA, cells were incubated with primary antibodies against LC3 and p62. Next, cells were stained with the Alexa Fluor 488-conjugated and Rhodamine-conjugated secondary antibodies (BD Biosciences), respectively. Nuclei were stained by DAPI (Sigma-Aldrich). Finally, images were taken under the fluorescence microscope (Leica, Wetzlar, Germany).

### Cellular viability assay

Cell viability was measured using 3-(4, 5-dimethylthiazol-2-yl)-2, 5-diphenyltetrazolium bromide (MTT) assay, cell counting kit 8 (CCK-8) assay, and EdU staining assay. MTT assay was performed using an *in vitro* toxicology assay kit based on MTT (Sigma-Aldrich) according to the manufacturer's instructions. CCK-8 (Dojindo, Kumamoto, Japan) was used to test cell viability. Briefly, cells were washed with PBS three times. Then a 1:10 diluted CCK-8 solution was added and incubated at 37°C for 2 h. The optical density was measured by microplate reader at 450 nm. For EdU staining assay, cells were permeabilized in 0.5% Triton X-100 in PBS followed by EdU staining with Click-iT EdU Alexa Fluor 488 imaging kit (Invitrogen) according to the manufacturer's instructions. Cells were examined by flow cytometry and data were analyzed using Cellquest software (BD Biosciences).

### Transmission electron microscopy

Observation of autophagosomes with transmission electron microscopy was performed as previously described (Hayashi et al., 2015). Cells were fixed with 2% glutaraldehyde in 0.1 M sodium cacodylate buffer (pH 7.2) for 1 h followed by 1% osmium tetroxide in 0.1 M sodium cacodylate buffer (pH 7.2) for 2 h. Fixed cells were blocked with 0.5% aqueous uranyl acetate overnight and treated with low-temperature dehydration and infiltration with a series of Epon/Araldite, then cells were embedded in 100% Epon/Araldite. Embedded cells were cut into 70 nm sections and stained with Reynolds' lead citrate. Cell sections were analyzed using a FFI Tecnai 12 transmission electron microscope.

### Statistical analyses

Statistical analyses were performed using GraphPad Prism 6.0 software. Data were analyzed by one-way ANOVA with multiple comparisons test (three or more data sets in a group) under Bonferroni correction. Results were expressed as mean±s.d. *P*<0.05 was considered statistically significant.
